# Educational robotics as a strategy for social inclusion and pedagogical intervention in vulnerable youth communities

**DOI:** 10.3389/frobt.2025.1662945

**Published:** 2025-12-01

**Authors:** Gustavo A. Acosta-Amaya, Juan A. Peña-Palacio, Jovani A. Jiménez-Builes

**Affiliations:** 1 GIS - Sustainable Engineering Research Group, Faculty of Engineering, Politécnico Colombiano Jaime Isaza Cadavid, Medellín, Colombia; 2 Information and Management, School of Administration, Universidad EAFIT, Medellín, Colombia; 3 Artificial Intelligence in Education, Department of Computer and Decision Sciences, Faculty of Mines, Universidad Nacional de Colombia, Medellín, Colombia

**Keywords:** educational robotics, vulnerable youth, school reintegration, pedagogical innovation, social inclusion, educational technology, artificial intelligence

## Abstract

**Introduction:**

In mining regions of Latin America, thousands of children and adolescents are deprived of formal education because of their participation in labor-intensive economic activities. This study addresses how educational robotics can serve as a strategy for both social inclusion and pedagogical intervention in communities with disrupted or nonexistent schooling.

**Methods:**

A multi-site intervention was implemented, directly benefiting 2,500 out-of-school or at-risk youth and 250 teachers in rural mining regions. The initiative encompasses the design and construction of educational robots and learning materials by university engineering students. Activities were conducted via project-based learning sessions and teacher training workshops. A mixed-methods approach was employed, integrating surveys, interviews, and participant observation to assess the impact on motivation, re-engagement with schooling, and pedagogical practices.

**Results:**

The findings indicated increased student engagement, enhanced collaborative learning, and a measurable rise in school re-enrollment within the participating communities. Educators reported enhanced confidence in utilizing technological tools and heightened motivation among students. The robots acted as mediating artifacts, facilitating dialogical, hands-on learning experiences and bridging gaps between formal education and local realities.

**Discussion:**

The results underscore the potential of educational robotics to serve not just as a pedagogical instrument but also as a transformative vehicle for fostering inclusion, motivation, and equity in marginalized environments. The initiative also demonstrates the significance of university-community collaboration in addressing educational inequality through innovation. Challenges include maintaining long-term impact and scaling the model to other contexts with similar vulnerabilities.

## 1 Introduction

In many mining areas in Latin America, children and adolescents are compelled to abandon their education or forgo enrollment due to the urgent necessity to provide economic support to their families. This phenomenon results in significant disruption to their educational and psychosocial development, perpetuating cycles of poverty, inequality, and social exclusion (Alam and Mohanty, 2024; UNICEF and ILO, 2021). Conventional educational initiatives have struggled to address the intricate realities of these societies. Consequently, it has been customary to marginalize such populations (Vargas and Pinos, 2022). In this setting, educational robotics (ER) has arisen as a novel approach to re-engage youth in learning and provide teachers with tools that address varied needs through participatory and inclusive pedagogies (Alimisis, 2013; Papert, 1980). School dropout poses a critical challenge in Latin America. Recent regional reports indicate that, on average, nearly 35% of Latin American youth do not complete secondary education (Arias Ortiz et al., 2024; OECD, 2024). This high dropout rate highlights significant gaps in access and completion, undermining the region’s social and economic development. In Colombia, the situation remains concerning. According to OECD data, approximately 22% of Colombian young adults (ages 25–34) have not completed upper secondary education (OECD, 2024). A notable geographic disparity also persists: rural areas exhibit significantly higher dropout rates than urban areas.

ER possesses a broad set of potentialities that position it as an innovative pedagogical strategy with social impact. First, it facilitates active and meaningful learning through authentic problem solving, experimentation and the concrete creation of knowledge, which stimulates both cognitive and socioemotional skills (Atman et al., 2023). In contexts of vulnerability, such as the mining territories addressed in this study, ER functions as a mediating artifact that transforms young people’s relationship with knowledge, fostering self-esteem, agency, and a sense of belonging. Moreover, it strengthens collaborative work, creativity and computational-logical thinking, which are essential competencies in today’s digital society (Alam and Mohanty, 2024; Sannicandro et al., 2022). From the teaching perspective, ER provides opportunities to innovate pedagogical practices, depart from conventional frameworks, and tailor teaching to the particularities of the context (Sanchez et al., 2024; Schina et al., 2020). Finally, its implementation through university-community partnerships enhances technology transfer processes while also addressing educational gaps and fostering inclusion and equity (Adel, 2024; Alimisis, 2021; Pavez et al., 2024).

This article focuses on exploring how ER, when implemented in rural mining territories, which are often marginalized, can serve as a social inclusion strategy and as a pedagogical intervention. The initiative examined herein targeted out-of-school youth and educators in mining communities, utilizing robotic kits designed and built from the ground up by university students, along with complementary didactic materials. Each robotic kit integrated programmable microcontrollers, proximity sensors, traction motors and modular constructions constructed from inexpensive and readily available materials, among others. The primary contribution of this study is to demonstrate a low-cost, scalable, and empirically grounded educational robotics program designed to promote social inclusion in rural mining regions. We deliberately selected commercial components and recycled materials to maximize affordability, ease maintenance, and foster local ownership—conditions essential in resource-constrained settings. Within this framework, we ensure technical transparency to enable replication (robot kits used), while the evaluation focuses on pedagogical and social outcomes (school re-engagement, motivation, and teaching practices). As a brief synthesis of related work, recent advances in time–frequency attention and tensor factorization offer promising strategies for analyzing multimodal educational data (Wang et al., 2025; Wang et al., 2025; Wang et al., 2025); however, such approaches fall outside the scope of the present work and serve as references for future studies.

Compared to traditional instruction, educational robotics offers a more active and participatory learning experience, which may lead to better academic outcomes. Several studies and meta-analyses have reported that robot-supported instruction tends to yield better learning outcomes than traditional lecture-based teaching (Wang et al., 2023). This effect arises because robots enable students to visualize and apply abstract concepts in practical ways, providing an active experience that supports a deep understanding of the subject. In contrast, traditional teaching often centers on passive content delivery, providing fewer opportunities to develop problem-solving and creative thinking skills (Ouyang and Xu, 2024). Nevertheless, educational robotics requires resources (equipment, software) and specialized teacher training, factors not always available in traditional settings, so implementation involves significant logistical challenges.

Regarding programming instruction, purely programming courses (for example, on-screen coding without robots) and educational robotics present distinct advantages and disadvantages. On the one hand, pure programming environments focus attention on algorithmic concepts without the added demands of handling hardware. Recent experimental studies have shown that elementary students who learned with a visual environment (for example, Scratch) without a robot achieved a firmer grasp of programming concepts than their peers who used the same environment with a physical robot (Sigayret et al., 2025). The presence of the robot introduced extra complexity and cognitive load, which may have hindered short-term conceptual learning (Sigayret et al., 2025). In exclusively digital programming courses, class time is dedicated entirely to coding skills, which can accelerate progress on foundational computing concepts.

On the other hand, educational robotics offers unique benefits: programming a tangible object that responds physically can increase motivation and give concrete meaning to abstract programming concepts (Ouyang and Xu, 2024; Sigayret et al., 2025). Many students find coding more engaging when a robot moves or solves a real-world challenge, which heightens interest and enjoyment (Wang et al., 2023; Sigayret et al., 2025). In addition, curricula have increasingly incorporated computational thinking, recognizing its value for framing and solving problems in ways that computers can execute (Gerosa et al., 2022; Yeni et al., 2024). While a traditional programming course maximizes efficiency in teaching syntax and logic, educational robotics trades some of that simplicity for greater motivation, practical context, and an interdisciplinary perspective (Tang et al., 2025; Lu et al., 2025). The choice between approaches depends on instructional goals: when the aim is rapid depth in programming concepts, a traditional course may be suitable; when the goal is sustained interest, creativity, and practical application, robotics adds value that software alone struggles to match (Díaz-Lauzurica and Moreno-Salinas, 2019).

Finally, when comparing educational robotics with other active methods, such as Project-Based Learning (PBL) without the use of robots, key similarities and differences also emerge. Both approaches share an active, student-centered philosophy, in which knowledge is built through practical projects and collaboration, as opposed to the traditional teaching approach that emphasizes memorization (Ouyang and Xu, 2024). Evidence shows that PBL, even without advanced technology, improves student motivation and engagement relative to passive methods (Valls Pou et al., 2022) by enabling meaningful work on real problems. In this sense, educational robotics can be viewed as a specialized modality within PBL, where projects involve building and programming robots. It therefore carries the general benefits of PBL, including collaboration, critical thinking, and problem solving. In particular, robotics naturally integrates digital and STEM (Science, Technology, Engineering, Mathematics) competencies: students plan and execute a project while also learning programming, electronics, and design, developing technical skills that are difficult to address in projects without technological components. This convergence of disciplines in a single robotics project can amplify educational impact, as demonstrated in studies where robotics has significantly enhanced students’ ability to apply theoretical knowledge to practical problems (Valls Pou et al., 2022). The novelty of working with robots often sparks additional curiosity and attracts students who might engage less with traditional projects, which supports inclusion and participation.

At the same time, using proven tools (robotics kits) is crucial because real risks arise in robotics workshops: sensor faults or code issues can consume time and frustrate participants if not correctly managed. Consequently, although traditional PBL and educational robotics share a common methodological core, the latter stands out by adding a richer technological context that can strengthen learning in STEM areas and 21st-century skills. Accordingly, educational robotics does not invalidate other approaches; it plays a complementary role. Educational robotics demonstrates particular value in domains such as computational thinking, creative problem-solving, and motivation toward the sciences, outcomes that are difficult to achieve with the same intensity through traditional methods or non-technological projects. The evidence reviewed suggests that, when implemented effectively, educational robotics combines the strengths of hands-on learning with technological immersion, providing a comprehensive educational experience that enriches and extends the benefits of conventional methodologies (Wang et al., 2023; Valls Pou et al., 2022).

Bravo et al. (2017) identify four primary purposes for utilizing ER kits as learning objects: to enhance the topic development of class sessions, to cultivate skill acquisition, to engage and motivate students in the learning process, and to enrich the teaching experience. The article is organized as follows: the subsequent chapter presents the materials and methods employed in the research design. Chapter three brings the results, while chapter four presents the main findings through discussion. Ultimately, conclusions and bibliographic references are provided.

## 2 Materials and methods

This chapter details the research design, participants demographics, ER interventions, as well as the methods for data collection and analysis. The research employed a mixed methods approach to examine the impact of ER pedagogical interventions for social inclusion in vulnerable youth communities. To facilitate adoption, external assessment, and replication, we document the project’s technical and procedural components in full. In particular, we provide the specifications of the robotics kits used during classroom activities to support adoption and maintenance in school communities. Complementing this technical documentation, the study reports educational outcome metrics using a pre–post design with standardized instruments, focusing on motivation for learning, self-efficacy in technology use, and school re-entry.

### 2.1 Research design

The research employed a quasi-experimental pretest-posttest design utilizing a single group, augmented by qualitative techniques for enhanced contextual understanding. The intervention was implemented in eleven mining communities in Colombia over the course of 1 year. To assess the intervention’s impact, pre- and post-process instruments were applied: (1) context-adapted motivation and self-efficacy scales, (2) school re-enrollment records, and (3) semi-structured interviews with educators and facilitators. Quantitative data were examined using related samples t-tests, and qualitative data were studied by open and axial coding within the grounded theory framework. This hybrid approach facilitated the measurement of significant changes and the interpretation of apparent transformations in learning dynamics, school engagement, and pedagogical practice. This design has been widely described in the classical methodological literature, particularly by Shadish et al. (2002), who outline its scope and the main threats to internal validity, including history, maturation, and attrition (Shadish et al., 2002). Accordingly, we interpret the results as temporal associations between the intervention and the observed changes, without asserting absolute causal inference. At the same time, to strengthen the study’s methodological grounding and reflect contemporary perspectives on these designs, Reichardt (2019) updates and nuances the potential of quasi-experimental studies in applied settings. In this view, such designs can be helpful in specific contexts when researchers minimize external factors that may bias the results (Reichardt, 2019). We define the independent variable as the implementation of the educational robotics program, which involves participation in weekly workshops on robot construction and programming, along with teacher training activities. The dependent variables are the indicators measured at two time points (before and after the intervention), specifically participants’ motivation toward learning and their self-efficacy in using technology, as reported by the participants.

### 2.2 Participants

The study involved 2,500 children and adolescents, aged eight to sixteen, who attended weekly ER workshops conducted at community centers (n = 2,500). Simultaneously, 250 educators underwent pedagogical training centered on the application of educational technology with an inclusive approach, to facilitate the future replication of the initiative.

### 2.3 ER interventions

**FIGURE 1:**
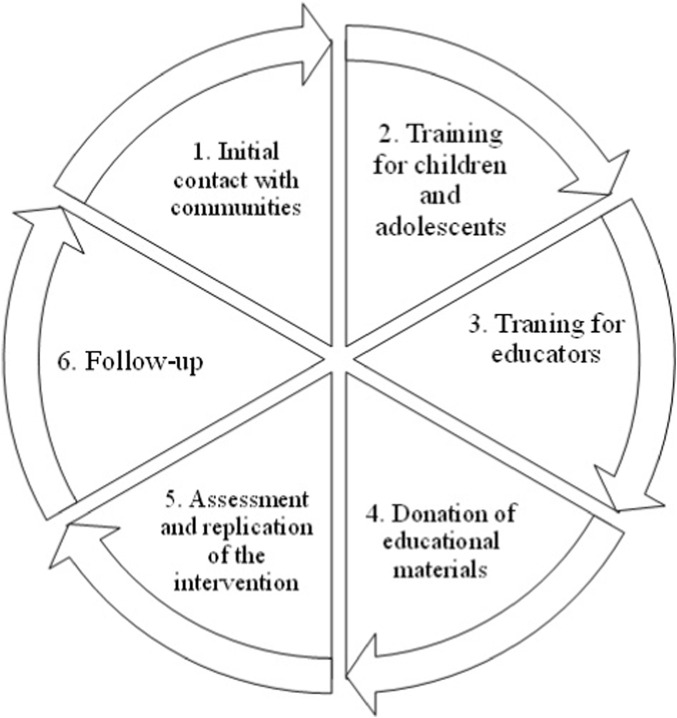
Cycle of the initiative’s intervention in the eleven rural communities affected by mining. Source: own construction.

The study entailed intervention in eleven communities through a cyclical process consisting of six distinct phases, as outlined below (refer to Figure 1).

#### 2.3.1 Initial contact with the rural communities of influence of the mining industry

The intervention began with a process of rapprochement and dialogue with the communities settled in the mining territories, prioritizing respect for local knowledge, territorial dynamics and the social actors involved. Exploratory trips and conversations with community leaders, educators, and grassroots organizations were conducted to ascertain specific needs and foster an environment of trust and co-responsibility. This phase was crucial for forming institutional alliances, culturally validating the content, and guaranteeing the logistical and pedagogical preparedness required for the initiative’s implementation.

#### 2.3.2 Training for children and adolescents

After establishing a connection with the communities, weekly workshops were designed and implemented for children and adolescents aged eight to sixteen, the majority of whom had either disengaged from the educational system or were considerably behind in their academic progress. The activities focused on the construction, manipulation, and basic programming of robots, employing a project-based methodology and collaborative learning (Barradas et al., 2024; Cardona et al., 2025). This training aimed to cultivate technical and cognitive skills while also fostering curiosity, collaboration, and self-confidence.

#### 2.3.3 Training for educators

A specific training component was developed for rural educators in the mining areas, aimed at strengthening their skills in the use of educational technologies and fostering inclusive practices. The training included theoretical and practical modules on ER, student-centered classroom strategies, and modification to the curriculum tailored to vulnerable contexts. Educators were supported in the planning and execution of sessions, enhancing their role as multiplier agents of knowledge and enablers of transformative experiences.

#### 2.3.4 Donation of robotic kits, books, guides and complementary educational material

To guarantee the continuity and sustainability of the intervention, robotic kits designed and assembled by university students were provided to each educational community, accompanied by culturally and linguistically adapted printed and digital materials. These kits comprised sensors, microcontrollers, transducers, modular components, user manuals, pedagogical guides, and activity booklets. The delivery included demonstration sessions, ensuring that both students and teachers understood its operation and didactic possibilities.

#### 2.3.5 Autonomous evaluation and replication of the intervention

Upon completion of the training phases, a participatory evaluation exercise was conducted, enabling children, adolescents, and teachers to reflect on the learning reached, the challenges faced and the prospects for future application. Educators were encouraged to autonomously replicate exercises through projects, adapting the content to different subjects and school levels. This phase aimed at fostering local appropriation of robotics as a sustainable educational tool, overcoming dependence on the external facilitation team.

#### 2.3.6 Follow-up

Finally, a technical and pedagogical follow-up system was established, including regular visits to the communities, virtual support from the university and the generation of forums for collective feedback. This follow-up made it possible to monitor the effective use of resources, provide assistance during technical difficulties and compile best practices to be shared in educational networks. It also served as a basis to assess the enduring effects of the intervention and to develop recommendations for future implementations of the model.

### 2.4 Data collection

#### 2.4.1 Quantitative data

Quantitative data collection was conducted using structured surveys at two intervals: prior to (pretest) and after (posttest) the intervention. The instruments were designed specifically for this study and include five-point Likert scales that measured variables including motivation for learning, perceived self-efficacy in technology use, and willingness to reintegrate into school. The surveys were conducted with a purposeful sample of 800 children and adolescents aged eight to sixteen, selected proportionally from the 11 participating communities. Administrative records about school reintegration and workshop participation were gathered as objective indicators of impact.

#### 2.4.2 Qualitative data

The qualitative information was acquired using semi-structured interviews, participant observations, and ethnographic field notes. Forty educators and ten community leaders were interviewed to gather insights regarding the learning experience, the use of robotics, and alterations in educational dynamics. The interviews were recorded and transcribed with the participants’ informed consent. Furthermore, project facilitators documented their observations of the learning environment, student engagement, and contextual adjustments implemented during the sessions on a weekly basis. Interviews were conducted in person, in private rooms at community centers, by two researchers trained in qualitative methods with local logistical support. The typical duration was approximately 45 min (with an approximate range of 30–70 min). This approach enabled us to capture social and pedagogical subtleties that enhanced the quantitative results.

#### 2.4.3 Instruments and measurement

This study analyzed two psychosocial scales: motivation toward learning and self-efficacy in technology use. “School re-entry” was not measured as a scale but as a behavioral outcome (yes/no). We administered questionnaires using a five-point Likert scale (1 = “strongly disagree,” 5 = “strongly agree”) at pretest and posttest.

##### 2.4.3.1 Motivation for learning

A brief five-item scale (contextually adapted from validated academic motivation instruments, based on the Motivated Strategies for Learning Questionnaire, MSLQ), assessing interest, perceived usefulness, effort, and collaboration in the workshops. Higher scores indicate greater motivation (Pintrich et al., 1991).

##### 2.4.3.2 Self-efficacy in technology use

A brief five-item scale (inspired by ICT self-efficacy measures) capturing confidence to program, connect sensors/actuators, troubleshoot errors, and learn new functions. Higher scores reflect greater self-efficacy (Compeau and Higgins, 1995).

The complete list of items appears in Appendix A.

### 2.5 Data analysis

#### 2.5.1 Quantitative analysis

The quantitative data were analyzed using IBM Corp (2021) SPSS Statistics software (version 28). Descriptive techniques were utilized to outline the sample and examine general trends. To assess the impact of the intervention, related samples t-tests were conducted comparing pre- and post-measurements on critical variables. A significance threshold of *p* < 0.05 was considered. Moreover, effect sizes (Cohen’s *d*) were calculated to assess the magnitude of the observed changes. The analysis of school re-enrollment data utilized relative frequencies and intercommunity comparisons. In addition to statistical significance, we reported effect sizes for the pretest–posttest comparisons, following conventional thresholds: approximately 0.2 = small, 0.5 = medium, and 0.8 = large. These benchmarks help contextualize the practical magnitude of the reported findings (Cohen, 1988; Lakens, 2013).

#### 2.5.2 Qualitative analysis

Qualitative analysis was conducted following a grounded theory approach (Charmaz, 2006), based on open, axial and selective coding. This approach centers on generating analytic categories from the data, using the constant-comparison method to contrast information segments within and across cases and time points (pre-post). Three researchers independently analyzed the transcripts, identifying units of meaning, developing categories, and common patterns within the narratives of educators, students, and community leaders. Subsequently, the information was triangulated with the field notes to increase the internal validity of the analysis. ATLAS.ti (2023) software was employed to structure and illustrate the conceptual networks generated from the interpretive process. The procedure involved a line-by-line reading to identify units of meaning, followed by grouping and relating categories and subcategories. It concluded with their integration into an explanatory framework aligned with the study’s objectives. The process was iterative: we refined and specified categories as new evidence emerged from interviews, observations, and field notes. To ensure consistency and traceability, three researchers independently coded a subset of transcripts and, through consensus sessions, agreed on a coding scheme that was then applied to the remaining material. We maintained analytic notes and a decision log throughout the process. We reinforced interpretive coherence by triangulating the emerging categories with the study’s quantitative results (motivation, self-efficacy, and school re-entry), so that the qualitative findings aligned with the patterns observed in the measurements.

#### 2.5.3 Evidence of validity and reliability

Expert review established content validity and guided item selection for rural contexts. The scales draw on previously validated instruments for academic motivation and digital self-efficacy (Pintrich et al., 1991; Compeau and Higgins, 1995). We assessed internal reliability using Cronbach’s alpha (Cronbach, 1951), which was estimated separately for the pretest and posttest. With the available data, motivation showed α = 0.974 (pre) and α = 0.945 (post); self-efficacy showed α = 0.965 (pre) and α = 0.964 (post).

### 2.6 Robotic kits

The robotic kits developed for the interventions consisted of four distinct types.

#### 2.6.1 Smart swarm

**FIGURE 2:**
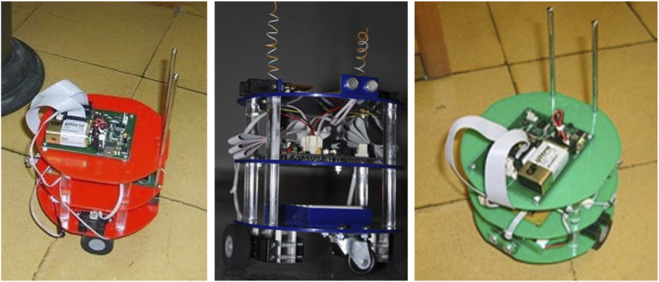
Swarm Smart robotic kit. Source: own construction.

The initial prototype consisted of a cooperative system composed of a swarm of three autonomous robots, programmed to move collaboratively within a previously mapped environment. The functional architecture drew inspiration from the collective behavior of bee swarms, with a central unit or “queen robot” that orchestrated the behaviors of subordinate devices, referred to as worker robots or beacon nodes. This model facilitated experimental tests centered on the efficiency of communication protocols and distributed coordination mechanisms among peripheral nodes (see to Figure 2).

In our implementation with commercial components, each colony unit used Arduino-class microcontrollers (ATmega328P, 16 MHz, 2 KB SRAM), HC-SR04 ultrasonic distance sensors (range 2–400 cm; typical accuracy ± 3 mm) and/or Sharp GP2Y0A21YK0F infrared sensors (10–80 cm), with short-range communication inspired by educational swarm platforms (proximity IR, as in Kilobot and e-puck). Traction ran on 18,650 Li-ion cells (≈3.6–3.7 V; 3,200–3,400 mAh). In classroom tests, the typical runtime was on the order of 1 h (depending on charge and duty cycle) under a moderate duty cycle, consistent with comparable educational platforms (≥1.5 h in e-puck). Sensor calibration protocols, CPU load, and technical specifications used in the field are available in each component’s technical documentation and in specialized literature (Arduino, n.d.; HandsOnTec, 2017; Sharp Corporation, 2006; Rubenstein et al., 2012; GCtronic/EPFL, 2007; Jones et al., 2018).

#### 2.6.2 Humanoid, swagway, rover, and others

**FIGURE 3:**
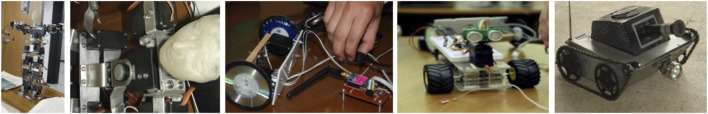
Humanoid robotic kit, Swagway, Rover and war tank. Source: own construction.

Undergraduate students from the systems, control, mechanical and geological engineering academic programs actively participated in the design and construction of several robotic prototypes, following a pre-stablished methodology. Notable advancements include a bipedal humanoid robot, including 23 direct current motors that facilitate sophisticated articulation. Similarly, a swagway was designed with bidirectional remote communication features and partially built from recycled materials. Notwithstanding its two-wheel configuration, the prototype exhibited exceptional stability during testing. A rover-type terrestrial vehicle was constructed, capable of wireless data transmission and reception, hence enhancing interaction possibilities in educational and experimental scenarios (see Figure 3).

The humanoid and “swagway” prototypes initially integrated mid-torque DYNAMIXEL AX-12A actuators (approximate stall torque 1.5 N·m at 12 V; 59 rpm; 0°–300°), evaluated only on an exploratory basis; instead, we used standard educational servos with stabilization via an MPU-6050 IMU (six-axis accelerometer/gyroscope) and L298N motor drivers (dual H-bridge, up to ∼2 A per channel) on a chassis powered by 18,650 Li-ion packs. The rover incorporated basic ranging (HC-SR04 ultrasound and/or Sharp IR) and 8/32-bit control electronics. For the self-balancing prototype, we followed recent references on two-wheel robots with IMU. We applied calibration tolerances following the examples in ROBOTIS (n.d.), TDK-InvenSense (n.d.), STMicroelectronics (2023), HandsOnTec (2017), and Sharp Corporation (2006).

#### 2.6.3 Arm and map generator

**FIGURE 4:**
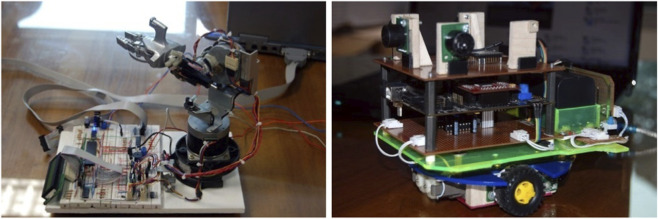
Robotic kit integrated with a navigation arm and map generator. Source: own construction.

During the prototype design phase of the project, a functional robotic arm was created by reusing components from a compact disc reader device. This gadget was accompanied by a computer application designed specifically to validate the programming algorithms presented during the teaching sessions. A graduate student developed an autonomous mapping robot capable of creating environmental representations without utilizing video cameras. This robot wirelessly provides environmental data to a desktop application, which processes it to generate a topographic survey of the affected surface (Acosta, 2019). Over time, this architecture evolved to implement a more sophisticated model based on the SLAM (Simultaneous Localization and Mapping) paradigm, which allows simultaneous localization and mapping of the environment, significantly expanding the autonomous navigation capabilities of the system (see Figure 4).

The arm utilized repurposed servomotors (linear mechanism from a CD drive) and modular, education-level controllers. The mapping robot employed camera-free distance sensing (ultrasonic/IR) and on-board processing based on an ESP32 microcontroller (Espressif Systems, 2025). In future configurations, a moderate-cost SBC (for example, Raspberry Pi 3 B+) may support on-board logging and processing. As a methodological baseline, the localization and mapping scheme follows the classical 2D SLAM (Simultaneous Localization and Mapping) approach. Lightweight LiDAR (for example, RPLIDAR A1, 0.15–12 m; 5.5–10 Hz) is reserved for future work subject to budget, while maintaining cost constraints and datasheet configurations (Sharp Corporation, 2006; HandsOnTec, 2017; Raspberry Pi Ltd, 2025; Cadena et al., 2016; SLAMTEC, n.d.).

#### 2.6.4 RoboED

**FIGURE 5:**
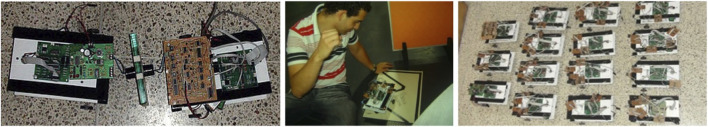
The RoboED robotic kit was the most frequently constructed. Source: own construction.

RoboEd comprises two acrylic plates assembled by bars of the same material, facilitating a modular and robust structure. The lower plate contains the locomotion mechanisms, which possess a flexible configuration that may be adjusted to teach fundamental principles such as mechanical stability. The central space, situated between the two plates, houses the electronic cards, connectors, and batteries, so promoting an organized and functional design. The upper plate facilitates the attachment of diverse transducers (sensors) via Velcro® strips, permitting swift customization. This platform also includes a set of connectors that allow the robot to operate autonomously based on the objectives defined by the children and teenagers throughout the sessions. Figure 5 illustrates that participants can concurrently integrate one or many sensors, depending on the desired interaction or robotic response they intend to develop.

The RoboED platform, with a modular acrylic architecture, used Arduino-class controllers (16 MHz, 2 KB SRAM) and a central bay for boards, connectors, and batteries, enabling interchangeable transducers (ultrasound and IR) and DC/servo actuators. This design philosophy aligns with widely used modular educational robots (for example, Thymio II and e-puck), which combine perimeter sensors, IMU, and open expansion for classroom activities. Assembly steps, firmware parameters, and performance metrics are available in the component datasheets (Arduino, n.d.; Riedo et al., 2013; GCtronic/EPFL, 2007).

## 3 Results

This section delineates the results obtained from the execution of the ER intervention, utilizing a mixed-methods research framework. A total of 800 children and adolescents were assessed from an initial group of 2,500 persons. Quantitative results were acquired using structured surveys conducted pre- and post-intervention, and qualitative data were collected through interviews, observations, and field notes. The results were structured in accordance with the research objectives, focusing on variables such as motivation, self-efficacy, school re-entry, and pedagogical transformation; however, we will first address the robotic kits that were designed and built, along with the instructional content conveyed.

### 3.1 Educational contents

Table 1 presents a selection of titles representative of the contents addressed by the ER strategy. Table 1 presents a compilation of titles that exemplify the subjects covered by the ER strategy. Although the initiative allows teaching a wide variety of subjects (including biology, geography, art, among others), it has been observed that children and adolescents show a significantly greater interest in technology-related contents, even when it comes to advanced topics. This tendency suggests a high receptivity to learning mediated by digital tools, underscoring the importance of integrating emerging technologies in non-conventional educational contexts.

**TABLE 1:** Some of the educational content taught.

Areas	Basic learning units
Electronics	Ohm’s Law
	Series of resistors
	Voltage divider
	DC electric motor
Sensors	Passive sensors: diode emitted lights (LED), resistor dependent lights (LDR), electrical switches, microphone
	Active sensors: ultrasonic, infrared, phototransistor, object reflector sensor
Mechanical physics	Rigid body equilibrium
	Friction
	Center of gravity
Algorithms	Structures: sequential, cyclic and logical decision structures
Other	Robot assembly
	Robot aesthetics
	Communications

Source: adaptation based on Jimenez et al. (2013).

### 3.2 Statistical evaluation of the research data

#### 3.2.1 Quantitative results

Data from 800 children and adolescents, chosen as a representative sample of the intervention population, were analyzed. Standardized instruments were utilized at two intervals (pretest and posttest), focusing on three dimensions: motivation for learning, self-efficacy in technology usage, and intention to re-enroll in educational institutions. The pre–post increases describe changes during the intervention period in the analyzed sample. In the absence of a comparison group, we do not interpret these results as conclusive evidence of causal effects; rather, we view them as consistent with an improvement temporally associated with the intervention (see Table 2).

**TABLE 2:** Synthesis of results.

Variable	Pretest (M ± SD)	Posttest (M ± SD)	*t* (799)	p-value	Cohen’s d
Motivation	3.2 ± 0.7	4.4 ± 0.5	24.16	<0.001	1.73
Self-efficacy	2.8 ± 0.6	4.1 ± 0.6	22.83	<0.001	1.58
School re-entry	-	38%	-	-	-

Source: own construction.

Motivation showed a significant increase: M = 3.2 (SD = 0.7) in pretest to M = 4.4 (SD = 0.5) in posttest, *t*(799) = 24.16, *p* < 0.001.

Self-efficacy went from M = 2.8 (SD = 0.6) to M = 4.1 (SD = 0.6), *t* (799) = 22.83, *p* < 0.001.

Thirty-eight percent of the students who were out of school at baseline initiated or completed school reentry processes at the end of the intervention.

These results indicate positive and significant effects of the intervention, especially in terms of reactivating school interest in contexts of high exclusion.

#### 3.2.2 Qualitative insights

The qualitative analysis included semi-structured interviews with 40 educators and 10 community leaders, in addition to field observations and facilitators’ logs. Three primary categories emerged from this corpus.

##### 3.2.2.1 Student empowerment

Young individuals conveyed a sense of empowerment, motivation, and pride in constructing and programming their own robots. Many described it as their first meaningful experience with formal knowledge.

##### 3.2.2.2 Teaching pedagogical innovation

Educators indicated a shift in their pedagogical approach, transitioning from transmissive methods to participatory, exploratory strategies adapted to their contexts.

##### 3.2.2.3 Community transformation

Community leaders and educators noted that the intervention reinforced intergenerational connections, fostered elevated educational aspirations, and acknowledged the university’s significance in traditionally marginalized territories.

These perceptions enhance the comprehension of the impact of ER beyond traditional indicators, highlighting its capacity to induce attitudinal and cultural transformations.

#### 3.2.3 Statistical summary

A synthesis of the most relevant quantitative results is presented below (see Table 3), and the context-adapted scales, grounded in widely used instruments, showed consistent pre–post patterns and high internal reliability (post α ≥ 0.94) for motivation and self-efficacy (see Table 4).

**TABLE 3:** Pretest–posttest change summary.

Variable	Δ (Post–Pre)	%Δ pretest	%Δ posttest	Post proportion
Motivation	1.20	37.50	27.27	-
Self-efficacy	1.30	46.43	31.71	-
School re-entry	-	-	-	38.00%

Source: own construction.

**TABLE 4:** Instrument characteristics and internal reliability (Cronbach’s α).

Scale	No. of items (k)	α (pre)	α (post)
Motivation	5	0.974	0.945
Self-efficacy	5	0.965	0.964

Source: own construction.

### 3.3 Interpretative analysis of the key variables

Data triangulation made it possible to establish that the use of ER had a significant impact on intrinsic motivation for learning, as well as on the participants’ perception of self-efficacy. The tangible, creative and collaborative nature of ER facilitated meaningful learning experiences, especially in contexts where traditional schooling conditions had been weakened by systemic poverty and child labor.

The intervention served as a catalyst for educational reflection and transformation among educators. Many indicated that they had included active methodologies into their daily practices, so expanding their pedagogical repertoire and enhancing student connection. This evolution was favored by the availability of materials, ongoing training, and close support from the university team.

The re-enrollment of 38% of out-of-school participants is both a measurable success and a testament to the transformative potential of contextualized educational technology in altering life paths. The connection among robotics, school belonging, and community empowerment confirms that innovation is not neutral; when focused on equity, it may be profoundly transformative.

## 4 Discussions

The findings of this study show that the implementation of ER as an intervention strategy in vulnerable youth communities had a positive and significant impact on the dimensions analyzed. In particular, improvements were observed in learning motivation, the strengthening of academic self-efficacy, an increase in school reincorporation, as well as transformations in educators’ pedagogical practices and in the perception of community agency. We interpret the findings as indicating temporal associations between the intervention and the changes observed in the sample, and we do not rule out the possibility of concurrent external factors. We confine our interpretation to the contexts and conditions in which the program was implemented, and we do not claim absolute causality without a comparison group. Even so, convergence with recent evidence in the educational robotics literature supports their empirical applicability.

In terms of motivation, quantitative results indicated an important increase in interest and willingness to learn, aligning with prior research linking the use of interactive technologies with the promotion of curiosity and emotional engagement in educational contexts (Alimisis, 2013; Bers, 2018). In this scenario, robotics served as a catalyst for reengagement with knowledge, offering youth entertaining, concrete, and collaborative experiences that enabled them to re-signify their relationship with knowledge.

A notable enhancement in the perception of competence to tackle academic assignments and resolve technical issues was observed regarding self-efficacy. This is explained by the active learning and project-based approach, wherein participants engaged with emerging technology and constructed functional prototypes. The possibility to design, program, and observe their own robots in action generated a sense of accomplishment that strengthened their confidence, which is particularly relevant in contexts where academic failure and stigmatization are prevalent.

The reintegration to school of 38% of the students who were initially out of the educational system is one of the most outstanding results of the study. This indicator signifies both a measurable enhancement and a symbolic one, reflecting the intervention’s capacity to influence life trajectories. The combination of technology, participatory methodologies and recognition of local knowledge fostered an environment that encouraged school re-enrollment and presented an alternate perspective on learning as a socially significant endeavor.

Concerning pedagogical innovation, the participating educators indicated a change in their didactic approaches, transitioning from transmissive models to more exploratory strategies centered on problem-solving and collaborative knowledge creation. This discovery aligns with research indicating that RE serves as a mechanism for curricular transformation when supplemented by teacher training and contextualized co-creation (Carbonaro et al., 2010). The gradual appropriation of technologies and the confidence cultivated by educators suggest that the proposal’s sustainability will mostly rely on ongoing institutional support and the curricular integration of these practices.

Finally, the qualitative results showed clear signs of community empowerment. The active engagement of university students, the construction of robots using recycled materials and the collaborative design of contents generated dynamics of technological appropriation within the communities. This community dimension allows transcending the instrumental rationale of technology, connecting it to social transformation processes from a perspective of educational and territorial justice.

This study used a single-group pretest–posttest design. We chose this approach for the initial phase, given the restrictive operational contexts (ethical and logistical constraints in rural settings). Because the study lacks a control group, we cannot entirely rule out concurrent external factors (for example, changes in community centers), which limits causal attribution of the observed changes. Accordingly, we interpret the changes as temporal associations consistent with the intervention and with the qualitative evidence collected, rather than as conclusive causal effects.

We interpret the results as effects associated with the integrated package, robot construction, programming, and teacher training, without attributing independent contributions to each component. This decision aligns with assessing the model’s feasibility and overall impact in high-vulnerability contexts with operational constraints. As a next step, we propose a component-wise evaluation suited to the context: (i) a stepped-wedge design with random sequencing of modules, for example, the gradual introduction of construction and/or programming, while maintaining teacher training as a foundation; or (ii) a two-factor, two-level factorial design: construction (yes/no) and programming (yes/no), with standardized teacher training held constant and a final phase in which all communities receive the full intervention.

We conducted quantitative analyses with 800 participants, drawn from a population of 2,500, who were selected based on availability in each community. The sampling invites the expansion of participant size and diversity in future phases, as well as the incorporation of selection procedures that enhance representativeness. The study took place in eleven rural communities with mining-related contexts; the next step is to test the model in other regions and compare results across population groups.

Follow-up occurred in the short term. Opportunities remain to conduct longitudinal evaluations that measure persistence of effects, school retention, and academic trajectories. Beyond changes in motivation and self-efficacy, future studies can integrate external indicators (for example, academic performance and persistence) and independent validations of re-entry records to strengthen the robustness of the findings.

Implementation conditions reflect the realities of rural settings with limited resources and connectivity, which constrained the variety of devices, tests, and metrics. Participation depended on the availability of both teachers and students, so the intensity and regularity of activities varied across communities. As an improvement, we propose modular and resilient protocols, offline materials, and staged strategies that reduce site-to-site variation and facilitate continuity.

The technological component used low-complexity kits and low-cost materials. The choice ensures access, but leaves the impact of more advanced configurations to be explored. A future line of work involves gradual pilots that incorporate new modules and compare results by technology level, while ensuring equitable access. Finally, university–community collaboration proved essential for execution; a next step is to formalize governance mechanisms and align the model with local policies to support scale and sustainability.

The feasibility of scaling depends on resources that vary across settings. Because unit costs fluctuate by context and market, and because this initial phase did not include systematic cost tracking, we do not report specific figures here. Instead, we assess return through observable educational outcomes in the short and medium term, such as school re-entry, gains in motivation, and self-efficacy. In subsequent phases, we suggest implementing standardized local cost collection to estimate simple indicators (for example, cost per participant, cost per re-entry) and to develop context-sensitive budget estimates and economic assessments.

Nevertheless, the study also revealed considerable challenges. The enduring sustainability of the intervention necessitates the institutionalization of the model, ongoing training for the teaching staff, and the integration of the evaluation and monitoring mechanisms with local educational policy. Furthermore, it is essential to broaden the scope of study to encompass additional social variables such as gender, ethnicity, or family involvement, which may exert a differentiated impact on the observed effects.

Building on our sociopedagogical orientation, future work may integrate time–frequency attention mechanisms, for example, wavelet-based methods, to handle nonlinearity, denoise low-cost multimodal logs and sensors, and surface engagement dynamics. In parallel, factor-based deep learning and channel-frequency attention can support dynamic weighting and multimodal fusion, while non-negative tensor factorization can yield interpretable patterns in collaboration and skill progression (Ke et al., 2024; Ke et al., 2024; Wang et al., 2025; Wang et al., 2025; Wang et al., 2025). These avenues align with recent literature, and researchers may pursue them while preserving affordability and feasibility.

In conclusion, when executed with pedagogical insight, cultural significance, and community engagement, ER emerges as a powerful strategy to enhance educational equity, motivation, student agency, and teacher transformation. This study contributes to consolidate a line of research that revalues the inclusive potential of technologies in historically marginalized contexts and raises new questions regarding the formulation of public policies that acknowledge and enhance these initiatives.

## 5 Conclusion

This study’s findings indicate that ER, when executed in a contextualized and participatory manner, holds great potential as a strategy for social inclusion and pedagogical transformation in marginalized youth communities. The intervention, carried out in Colombian mining territories, successfully stimulated the interest and motivation of children and adolescents excluded from the educational system, while also facilitating their reintegration into academic environments and enhancing the pedagogical skills of local educators.

In quantitative terms, significant improvements in learning motivation, technological self-efficacy and school re-enrollment rates were evidenced. In qualitative terms, relevant transformations emerged in teaching practices, collaborative work among students and the perception of ER as a meaningful, culturally relevant and pedagogically valuable tool. These transformations were facilitated by several factors: community ownership of the project, the university-territory articulation, the design of resources adapted to the context and a student-centered pedagogical approach.

The gradual implementation ensures both the sustainability of the process and its autonomous replicability by the participating schools. The provision of robotic kits and supplementary materials, along with educator training, strengthened local capacities to integrate technology in a sustainable and meaningful manner.

Nonetheless, significant obstacles persist. The sustainability of this type of intervention necessitates governmental policies that endorse educational innovation in marginalized contexts, with adaptable curricular frameworks that recognize and appreciate local knowledge. It is advisable to extend studies to longitudinal evaluations to assess the long-term impact of ER on students’ academic, emotional, and social development.

This study provides empirical and conceptual evidence about the transforming role of ER in scenarios of exclusion. In addition to serving as a technological instrument, it emerges as a mechanism to rebuild opportunities, democratize access to knowledge, and dignify the educational experience of individuals previously marginalized by the system.

## Data Availability

The original contributions presented in the study are included in the article/supplementary material, further inquiries can be directed to the corresponding author.

## Appendix A: Scale Items

General instruction: Indicate your level of agreement with each statement (1 = strongly disagree, 2 = disagree, 3 = neither agree nor disagree, 4 = agree, 5 = strongly agree).

### A.1. Motivation toward learning (5 items)

1. I am excited to learn new things during the workshops.
2. What I learn here is useful in my life.
3. I make an effort to complete activities even when they are difficult.
4. I enjoy working in teams to solve challenges.
5. I am interested in continuing to learn about robotics and technology.

### A.2. Self-efficacy in technology use (5 items)

1. I can program basic instructions so the robot completes a task.
2. I can connect and use sensors or actuators with minimal help.
3. When an error occurs, I can try different solutions until I fix it.
4. I can explain to someone else how to use the robot or the software.
5. I feel capable of learning new robot functions on my own.

Note: The items reflect an adaptation based on instruments used in the literature (MSLQ-type motivation and ICT self-efficacy). Experts reviewed and refined the items before fieldwork.
